# The closer, the better? The impact of generational residential distance on older adults’ mental health: evidence from China

**DOI:** 10.3389/fpubh.2026.1829724

**Published:** 2026-07-22

**Authors:** Shuaishuai Feng, Chengyue Wang, Shandong Zhou

**Affiliations:** 1 School of Public Administration, Hunan Normal University, Changsha, China; 2 School of Philosophy and Historical Culture, Xiangtan University, Xiangtan, China

**Keywords:** contact-based emotional support, older population, financial support, intergenerational residential distance, mental health

## Abstract

**Introduction:**

Against the backdrop of China’s accelerating population aging, the geographic separation of older adult parents from their adult children has become increasingly common. While intergenerational proximity is widely assumed to benefit older adults’ well‑being, empirical evidence on the optimal distance and its underlying mechanisms remains inconclusive. This study investigates the direct and indirect effects of intergenerational residential distance on the mental health of Chinese older adults, with a particular focus on the mediating roles of financial support and contact‑based emotional support.

**Methods:**

We utilized data from the 2023 China Longitudinal Study of Aging Social Survey, a nationally representative sample of community‑dwelling older adults. Intergenerational residential distance was categorized into four levels: cohabitation, same village/neighborhood committee, same city (different neighborhood), and different cities/provinces. Mental health was measured using a validated composite scale of depressive symptoms and life satisfaction. Mediation analyses were conducted via structural equation modeling, controlling for sociodemographic characteristics, health status, and social network variables.

**Results:**

Contrary to the conventional proximity assumption, older adults whose children lived in the same city (but not in the same household or immediate neighborhood) reported significantly better mental health than those in cohabitation or very close proximity. The mediation analysis revealed that children’s financial support exerted a robust positive mediating effect, whereas contact‑based emotional support showed a negative mediating effect. Notably, the negative indirect effect through emotional support was substantially larger in magnitude than the positive indirect effect through financial support, offsetting the total benefit of proximity.

**Discussion:**

These findings suggest that a moderate degree of intergenerational separation—sufficient to reduce daily frictions and role conflicts, yet close enough for practical assistance—may be more conducive to older adult mental health than over‑closeness. The stronger negative mediation via emotional support implies that frequent in‑person interactions, when accompanied by intergenerational disagreements or excessive caregiving demands, can undermine psychological well‑being. For older adults with adequate self‑care capacity, maintaining a moderate residential distance appears to preserve emotional bonding while minimizing daily conflict. Policy implications include the need for differentiated community‑based services that cater to diverse living arrangements, alongside targeted financial subsidy programs to offset the costs of separate residence, thereby supporting healthy and active aging in China’s rapidly changing demographic landscape.

## 1 Introduction

The predominant residential pattern in traditional Chinese society was multigenerational cohabitation. Under this arrangement, adult children living with their parents typically bear the primary responsibility for older adult care. Simultaneously, traditional ethical concepts rooted in Confucianism exerted a profound disciplinary influence on individual behavior, enabling the intergenerational cohabitation model to persist widely and enduringly. Confucian thought not only emphasized reverence for older adults but also explicitly stipulated the moral obligation for children to live with their aging parents and fulfill their duty of support (1, 2). Thus, the intergenerational cohabitation family structure effectively aligns with the older population’s care needs, making the family the cornerstone of the eldercare service system. Consequently, within the family sphere, the residential arrangements of the older adults directly determine their access to family support and social resources, significantly impacting their mental health (3). However, with the deepening of social modernization, the acceleration of population aging, and the dramatic transformation of family structures, the number of “empty-nest families” composed of older adult couples or solitary seniors continues to rise (4). Living apart from adult children has become the norm for the older population, leading to an increasingly severe imbalance between the supply and demand for eldercare. Potential financial disputes and emotional estrangement between older adult parents and their children can easily evolve into significant triggers for negative emotions on both sides.

According to the China Elderly Quality of Life Development Report (2019) (43) released by the China Research Center for Aging, only 33% of older adult people nationwide self-assess their health as good. Mental health not only directly affects the older population’s attitude toward life and satisfaction but also influences their physical health through mind–body interaction mechanisms, ultimately determining the overall quality of life in their later years. As a relatively vulnerable group with poorer health in society, the older population, especially the very old, face dual challenges to their physical and mental well-being. Entering old age, individuals must not only cope with the natural decline of physiological functions but also adapt to profound shifts in family and social roles. Failure to meet their material security and emotional comfort needs can easily trigger various mental health issues. Given this, why should intergenerational living arrangements be prioritized? This is because seniors’ housing arrangements not only determine their access to and utilization of eldercare resources (5), but also significantly influence their perceived convenience and comfort (6). Therefore, they profoundly impact their mental health. This logic reveals that older population housing arrangements directly constrain the effectiveness of children’s support for their parents, with the level of “upward support” provided by children emerging as a key determinant of the older population’s mental health.

Currently, as China’s society continues to age, the trend of older adult parents living apart from their children is becoming increasingly pronounced. Against this backdrop, the residential distance between children and their older adult parents not only directly constrains the accessibility of support behaviors such as emotional comfort, financial provision, and daily care, but also profoundly impacts the mental health of the older population. Therefore, in-depth exploration of the impact of intergenerational residential distance on the mental health of the older population and its underlying mechanisms holds significant theoretical and practical importance. This research not only helps accurately grasp the mental health landscape of the older population across different residential distances and clarify the mediating effect of children’s “upward intergenerational support,” but also provides scientific evidence for enhancing the quality of life in old age and promoting the high-quality development of China’s healthy aging initiatives.

While this study is situated in the Chinese context, its theoretical implications extend beyond a single country. China is undergoing a rapid demographic and familial transition—from traditional multigenerational cohabitation to diversified living arrangements—making it an ideal “critical case” for testing and refining theories of intergenerational relations. Specifically, the coexistence of deep-rooted filial norms and accelerating social mobility allows us to examine how residential distance simultaneously triggers both solidarity (financial compensation) and conflict (emotional friction). These dynamics speak directly to broader debates in aging societies worldwide: as living-apart becomes the norm in many post-industrial societies, understanding how families balance autonomy and support, and how cultural contexts moderate this balance, becomes a pressing global issue. Thus, the Chinese experience offers valuable comparative insights for other rapidly modernizing societies grappling with similar transformations in family elder care.

## 2 Literature and hypotheses

### 2.1 The impact of intergenerational residential distance on the mental health of older adults

Academic discourse on this topic has crystallized into two major theoretical paradigms: the “intergenerational solidarity model” and the “intergenerational conflict model.” Bengtson and Roberts (7) proposed the intergenerational solidarity model based on exchange theory, emphasizing that cohesion and consistency in family interactions are crucial for maintaining family stability and enhancing the well-being of older adults. In their view, spatial proximity facilitates intergenerational interaction, helping older adults access more adequate care resources and emotional support. However, the solidarity theory fails to fully explain the widespread tensions and conflicts within intergenerational relationships. Amid rapid societal transformation and widening value gaps, intergenerational conflicts within families have become increasingly prominent. Connidis and Mcmullin (8) noted that the solidarity model overlooks the pervasiveness of conflict in family interactions. Clarke et al. (9) categorized intergenerational conflict into six specific forms: communication styles, lifestyle habits, parenting philosophies, work attitudes, political ideologies, and family norms. Building on this, Lüscher and Pillemer (10) further developed the “Ambivalence Theory” of intergenerational conflict. This theoretical perspective offers a new interpretive pathway for understanding how intergenerational living arrangements impact older adults’ mental health: When older adults live in close proximity to their children, they gain greater emotional comfort and daily care, yet differences in lifestyle and values become more exposed, intensifying friction and psychological stress. Waite and Hughes (11) found that parents living with their children actually reported poorer health than those living apart. This stems from cohabitation patterns more readily triggering issues like lifestyle differences and intergenerational role conflicts, thereby increasing older adults’ risk of depression (12).

With rapid socioeconomic development and accelerated urbanization, China’s family structure has undergone fundamental changes. Economic growth has prompted younger generations to increasingly prefer independent living, while frequent population migration and job mobility have further widened the physical distance between parents and adult children (2). Concurrently, the preference for independent living among the older population has also significantly strengthened. Data indicates a sharp rise in the proportion of Chinese seniors living with their spouses, while cohabitation with adult children has declined substantially (12). Furthermore, the emergence of new online cultures and social formats has intensified generational gaps in life experiences and value systems. This has created greater communication barriers between children and parents, thereby increasing the risk of intergenerational conflicts (13). Research indicates that absolute obedience to parental wishes and the continuation of the family line are no longer the dominant cultural norms in Chinese family life (1, 14). These factors collectively weaken the support efficacy of traditional intergenerational cohabitation models for older population’s care.

Typically, we expect support from family members, and when they fail to provide it, our expectations are violated. Moreover, the impact of such negative interactions on mental health may be greater than that of positive interactions (15). Wenger et al. (16) found that older adults whose children live away enjoy greater freedom and autonomy, with more time to socialize with friends and participate in community activities. Living alone does not lead to complete isolation; rather, it provides opportunities to rebuild social connections and rekindle interests (17). Furthermore, living with adult children or maintaining close proximity through neighboring residences may lead to excessive reliance on their emotional or financial support. This dependency can amplify feelings of worthlessness and contribute to cognitive decline (12). Conversely, even when children do not reside with or near their parents, regular visits and online communication can reduce depressive symptoms and enhance cognitive function (18). Therefore, this paper argues that, compared to traditional intergenerational cohabitation or highly contiguous neighborhood living arrangements, maintaining an appropriate distance from adult children—a distance that ensures convenient access to support while satisfying older adults’ desire for autonomy—may be more conducive to their mental health development. Based on this, the following hypothesis is proposed:

*H1*: Compared to having children residing within the same household or village/neighborhood committee, older adults individuals whose children live within the same city exhibit higher levels of psychological well-being. We interpret this pattern as suggestive evidence that a moderate residential distance may be more conducive to mental health than co-residence or very close proximity.

### 2.2 The mediating role of intergenerational support from children

Social support was initially defined as a process of resource exchange between providers and recipients aimed at improving the recipient’s health and well-being (19). In health research, social support theory primarily explains its impact on individual mental health through two mechanisms: First, the Main-Effect Model. This model emphasizes that social support itself has a direct positive effect on mental health. That is, regardless of whether an individual is in a stressful situation, increased social support can enhance their mental health and optimize emotional experiences. Second, the buffering or protective mechanism. This mechanism suggests that when individuals face stress or adverse events, social support can effectively alleviate psychological burdens, thereby exerting a protective effect (20). For the older population, family support, as one of the core sources of social support, exerts a unique and profound influence on their mental health (21).

Based on the preceding discussion of the “intergenerational solidarity model” and the “intergenerational conflict model,” it can be observed that “spatial distance,” as an independent variable, simultaneously triggers “two opposing forces.” In short, the former emphasizes the positive role that proximity in living arrangements plays in promoting mutual assistance and communication, while the latter highlights the potential costs that close relationships may entail in terms of friction. To resolve this theoretical dichotomy, this study adopts a “balanced ecological perspective.” The net effect of residential distance on mental health depends on the dynamic balance between these two opposing forces. This framework not only reconciles existing theories but also provides a clear theoretical basis for hypotheses regarding the mediating roles of economic support (compensation mechanism) and emotional support (friction mechanism). Specifically, the impact of intergenerational residential distance on older adults’ mental health is twofold. On the one hand, closer proximity enhances children’s emotional support for their older adult parents, thereby improving the older adults’ mental health—a finding consistent with the solidarity model. On the other hand, living in close proximity increases the likelihood of intergenerational friction, which aligns with the predictions of conflict theory. Therefore, with appropriate spatial living apart, children tend to improve their older adult parents’ mental health through financial compensation.

Intergenerational residential distance is a key contextual variable constraining older adults’ access to financial support from their children. It not only affects the availability of intergenerational support but also determines its effectiveness. Based on the exchange motivation theory of intergenerational transfers, financial support received by parents from their children can be viewed as a return on the early care and resource investments made in their children (22, 23). Existing research indicates that children’s financial support for parents exhibits a pronounced “long-distance compensation” pattern. For instance, Li (24) found that increased intergenerational residential distance often prompts children to provide financial support for their parents. The underlying mechanism is that in long-distance living situations, children tend to compensate for the absence of daily care and emotional connection through material support (25). This suggests that as the physical distance between generations increases, children may strengthen intergenerational bonds through financial transfers to compensate for the absence of companionship and care. This phenomenon confirms the existence of “ritualistic giving.” Ritualistic giving refers to children’s financial support for their parents being more of a symbolic expression, intended to demonstrate their recognition of filial obligations (26). Based on this, the following hypothesis is proposed:

*H2a*: Financial support from children plays a positive mediating role in the impact of intergenerational residential distance on older adults' mental health levels. Specifically, the greater the intergenerational residential distance, the higher the likelihood that children will provide financial support to their parents, thereby exerting a positive mediating effect on older adults' mental health.

Beyond financial support, emotional support is also a crucial safeguard for maintaining the mental health of older adults. Its core lies in children providing spiritual comfort and psychological stability to their parents through communication, understanding, and emotional exchange. Emotional support profoundly reflects the strength of the emotional bond between parents and children. Numerous studies indicate that, compared to financial provision or daily care, emotional support has the most significant positive effect on the mental health of older adults (27). Gopalakrishnan et al. (28) found that a supportive, caring, and warm atmosphere fostered among family members and friends not only enhances the quality of life for older adults but that such positive emotional interactions may also promote their cognitive function. The physical distance between parents and children significantly influences the extent to which parents receive emotional support from their offspring (29). When older adults individuals reside near or with their children, they are more likely to gain understanding and care through frequent face-to-face interactions, leading to markedly higher life satisfaction and happiness. Conversely, excessive distance reduces communication opportunities, weakens emotional bonds, and may induce loneliness and emotional detachment. As China’s family relationship patterns evolve, parental needs are shifting from material support toward emotional fulfillment, placing increasing emphasis on intergenerational communication, trust, and mutual understanding. Based on this, the following hypothesis is proposed:

*H2b*: Children's contact-based emotional support plays a negative mediating role in the impact of generational residential distance on older adults' mental health levels. Specifically, the greater the generational residential distance, the less likely children are to provide contact-based emotional support to their parents, thereby exerting a negative mediating effect on older adults' mental health.

## 3 Data and variables

### 3.1 Data source

Before proceeding with the research, it is necessary to define the study population, “the older population.” Currently, there are two primary age criteria for defining the older population: the first categorizes individuals aged 65 and above as older population, primarily applied in developed countries or regions; the second defines those aged 60 and above as older population, predominantly used in developing countries or regions. China typically adopts the second criterion, designating individuals aged 60 and above as older population. For the purposes of this study, the older population are defined as residents aged 60 and above who hold Chinese household registration.

The data used in this paper are sourced from the 2023 China Longitudinal Study of Aging Social Survey (CLASS 2023). CLASS is a large-scale, nationwide, and continuous social survey project designed and implemented by the Institute of Gerontology at Renmin University of China. CLASS selects individuals aged 60 and above from household units as its sample subjects, meeting the criteria for this study’s research population. The CLASS 2023 data encompassed a baseline survey covering 28 provinces and municipalities nationwide (excluding Hainan, Tibet, and Xinjiang), collecting a total of 11,670 samples. This sample size sufficiently supports the research conducted in this paper, requiring only the removal of missing values across various variables. It should be noted that the independent variable in this study is generational residential distance—specifically, the distance between the older population’s person’s primary residence and the primary residence of their closest adult child, as defined by administrative boundaries. The fundamental requirement for inclusion in this variable is that the older adults person must have at least one living child over the age of 18. The survey questionnaire categorized intergenerational residential distance into eight levels: same household, same village/neighborhood committee, same subdistrict/township, same district/county, other districts/counties within the same city, other cities within the same province, other provinces, and overseas. Among these, the latter three categories (other cities within the same province, other provinces, and overseas) represent long-distance intergenerational residence, each accounting for less than 2% of the total sample. Therefore, this study focused on detailed analysis within close proximity, selecting five categories—same household, same village/neighborhood committee, same subdistrict/township, same district/county, and other districts/counties within the same city—as the primary research focus. Ultimately, 8,754 samples were included in the study and analysis.

### 3.2 Variable measurements

The primary variables addressed in this paper are: mental health, intergenerational residential distance, financial support from children, and contact-based emotional support from children. The indicators are described as follows:

#### 3.2.1 Mental health

Following the methodology of Tao and Shen (30), the mental health level score is calculated by summing the depression score, life satisfaction score, and cognitive function score. The operationalization is as follows:

(1) Depressive mood was measured using the Center for Epidemiologic Studies Depression Scale (CES-D), a widely adopted instrument both domestically and internationally, which demonstrates good reliability and validity (27). This scale comprises nine items assessing older adults’ mood, loneliness, eating habits, sleep, sense of worth, and life circumstances. Six negative items are reverse-scored from 1 to 3 based on responses of “never,” “sometimes,” and “often,” while the remaining three positive items are scored directly from 1 to 3 (3). Samples with “unable to answer” responses were treated as missing values and excluded. The sum of scores from the nine questions constitutes the depression scale score, ranging from 9 to 27 points. Higher scores indicate better mental health. The scale’s Cronbach’s alpha coefficient is 0.7, demonstrating good reliability; (2) Life satisfaction was measured using item B17: “Overall, are you satisfied with your current life?” Response options—"Very satisfied, Somewhat satisfied, Neutral, Somewhat dissatisfied, Very dissatisfied”—were reverse-scored from 1 to 5 points; (3) Cognitive function is assessed using the Mini-Mental State Examination (MMSE) developed by Folstein et al. (31). This scale includes questions across five dimensions: time orientation, place orientation, immediate recall, delayed recall, and calculation ability. Key questions include: “Correctly state the date of National Day (year, month, day)”; “Correctly state the name of your residential community or village and the current President of the country”; “Perform five consecutive calculations of 100 minus 7.” Each of the 16 questions is worth one point. The total score ranges from 0 to 16, with higher scores indicating better cognitive function. The scale demonstrates good reliability, with a Cronbach’s alpha coefficient exceeding 0.7.

#### 3.2.2 Intergenerational residential distance

The core explanatory variable in this study is intergenerational residential distance. Measured using responses to the question “Where does this child currently reside?” In the CLASS2023 dataset, answers were categorized into two groups: “Same household” and “Same village/neighborhood committee.” They were assigned a value of 0, indicating cohabitation or proximity within the same household or village/neighborhood committee (i.e., intergenerational co-residence or adjacent residence); “Same subdistrict/township,” “Same district/county,” and “Other districts/counties within the same city” were grouped and coded as 1, indicating the child resides within the same city. Drawing from prior research, intergenerational residential distance is measured by the distance between the older population’s person’s primary residence and the nearest child’s primary residence among those not cohabiting with their children (32).

#### 3.2.3 Financial support from children

This question measures children’s financial support for older adult parents: “Over the past 12 months, has this child provided you (or your surviving spouse living with you) with money, food, or gifts?” What was the total value of these financial contributions? For the amount ranges in the options, the median value is assigned as the fixed numerical value for that option. For example, the option “1–199 yuan” is assigned a value of “100 yuan.” The total financial support from all surviving children is summed and divided by the total number of children who answered this question to obtain the average financial support amount. Taking the logarithm of this average yields a score ranging from 0 to 10, where a higher score indicates stronger financial support from children to their older adult parents (33).

#### 3.2.4 Emotional support from children (a proxy based on contact frequency)

Following common practice in large-scale survey research (34), this variable is primarily measured by the frequency of contact (via phone, internet, or in-person) and visits between children and their parents over the past 12 months. We acknowledge that contact frequency is an imperfect proxy for the quality or emotional depth of support; however, it captures the behavioral manifestation of emotional bonding and is widely used in the literature. The options for contact frequency and visit frequency—"almost daily, at least once a week, at least once a month, several times a year, and rarely (including ‘no need for contact’)”—are each assigned reverse scores ranging from 1 to 5. The sum of these two scores constitutes the contact-based emotional support score for each individual child. The contact-based emotional support scores from all surviving children are aggregated and divided by the total number of children responding to this question to yield the average child contact-based emotional support score, ranging from 2 to 10.

The control variables include: gender, age, educational attainment, marital status, household registration status, income, self-rated health, assessment of basic functional abilities, and assessment of activities of daily living, etc.

Table 1 presents descriptive statistics for the variables included in the model. Overall, the mean mental health score for older adult parents as the dependent variable was 37.978, indicating that parents’ mental health generally falls within the upper-middle range. Regarding the independent variable of intergenerational residential distance, 60.0% of older adults individuals resided within the same household or village/neighborhood committee as their children, while 40.0% resided within the same city. This indicates that co-residence and proximity living remain the predominant choices among the sample. Regarding the mediating variables, the mean logarithm of children’s financial support was 6.529, while the mean of children’s contact-based emotional support was 7.238, both indicating an upper-middle level.

**Table 1:** Descriptive statistics of the core variables in this study (N = 8,754).

	Variable name	Mean	SD	Minimum	Maximum
Dependent variable	Mental health	37.978	4.740	18	48
	Depressive mood	20.359	3.301	9	27
	Life satisfaction	3.810	0.737	1	5
	Cognitive function	13.809	2.478	0	16
Independent variable	Intergenerational residential distance	0.400	0.490	0	1
	This household and this village/neighborhood committee	0.600			
	This prefecture-level city	0.400			
Mediator variable	Financial support from children	6.529	2.261	0	9.9
	Emotional support from children	7.238	1.556	2	10
Control variable	Gender	0.514	0.500	0	1
	Female	0.486			
	Male	0.514			
	Age	71.593	5.978	60	98
	Education	2.340	0.915	1	4
	No formal education	0.182			
	Elementary school graduate or below	0.423			
	Junior high school graduate	0.270			
	High school graduate or above	0.125			
	Marital status	0.830	0.376	0	1
	Not in a marital relationship	0.170			
	In a marital relationship	0.830			
	Hukou	0.586	0.493	0	1
	Non-agricultural hukou	0.414			
	Agricultural hukou	0.586			
	Personal income (log-transformed)	4.123	0.511	2	5.38
	Self-assessment of health	3.542	0.778	1	5
	Very unhealthy	0.009			
	Somewhat unhealthy	0.072			
	Average	0.367			
	Somewhat healthy	0.473			
	Very healthy	0.079			
	Basic activity assessment	8.240	1.052	8	24
	Daily activity assessment	6.310	1.267	6	18
	Total number of living children	2.268	1.123	1	9
	Pension security	1.155	0.559	0	6
	Social support	14.665	4.869	0	30
	Internet usage	0.489	0.500	0	1
	No use	0.511			
	Use	0.489			
	Community environment	3.462	3.712	0	16
	Social engagement	1.954	3.975	0	25
	Work environment	2.869	0.959	1	5
	Very poor	0.079			
	Fairly poor	0.227			
	Average	0.509			
	Fairly comfortable	0.114			
	Very comfortable	0.070			
	Current work frequency	1.891	1.497	1	5
	Not participating	0.706			
	Several times a year	0.042			
	At least once a month	0.036			
	At least once a week	0.088			
	Almost every day	0.129			
	Retirement status	0.408	0.491	0	1
	No	0.592			
	Yes	0.408			

### 3.3 Statistical model

The dependent variable in this study is the mental health level of older adults, which is a continuous variable ranging from 18 to 48 points. It is calculated by summing the scores of three indicators: depressive mood, life satisfaction, and cognitive function. For quantitative research with a continuous dependent variable, this paper first employs ordinary least squares (OLS) for model construction and analysis. The regression model is as follows:


$$\begin{array}{l} Y_{{\text{mentalhealth}}_{i}}=Y_{{\text{mentalhealth}}_{i}}=\beta_{0}+\beta_{1}X_{{\text{distance}}_{i}}+\\ \beta_{2}X_{{\text{financialsupport}}_{i}}+\beta_{3}X_{{\text{emotionalsupport}}_{i}}+\\ \beta_{4}+{\text{Control}}_{i}\varepsilon_{i} \end{array}$$

In the above equation, $Y_{{\text{mentalhealth}}_{i}}\;$ is the dependent variable, representing the mental health level of the i-th individual. $X_{{\text{distance}}_{i}}$ is the independent variable, indicating the intergenerational residential distance of the i-th individual. It is a dichotomous variable, with the reference category being “residing in the same village/neighborhood committee as the household.” $X_{{\text{financialsupport}}_{i}}$ denotes the financial support received by the i-th individual from their children, while $X_{{\text{emotionalsupport}}_{i}}$ indicates the contact-based emotional support received by the i-th individual from their children. Both are continuous variables. $\beta_{0}$ represents the constant term, $\beta_{1},$ $\beta_{2},$ $\beta_{3},$ $\beta_{4}$ are the parameters to be estimated, ${\text{Control}}_{i}$ is a set of control variables, $\varepsilon_{i}$ is the random error term.

When the independent variable is a dichotomous variable, and both the mediating variable and the dependent variable are continuous variables, the mediation effect analysis should employ the Bootstrap method (35). Therefore, to examine the mediating role of intergenerational residential distance in the relationship between children’s financial support and contact-based emotional support and older adults’ mental health, this study will employ the Bootstrap method to conduct significance tests and analyses of the mediating effects of children’s financial support and contact-based emotional support.

## 4 Analysis results

### 4.1 Baseline regression results

Older population’s mental health is treated as a continuous variable, thus analyzed using a multiple linear regression model. Table 2 presents the OLS model regression results: Model 1 examines the impact of intergenerational residential distance on older population’s mental health without any control variables; Model 2 incorporates control variables based on Model 1; Model 3 further includes mediating variables to test the effects of children’s financial and contact-based emotional support on older population’s mental health. Multicollinearity checks on core explanatory and control variables revealed variance inflation factors (VIF) below 4.5, with an average VIF of 1.942, confirming the absence of multicollinearity issues. The model passed significance testing.

**Table 2:** OLS regression results.

Variables	Mental Health		
	Model 1	Model 2	Model 3
Intergenerational residential distance (reference group: this household and this village/neighborhood committee)			
This prefecture-level city	1.519***	0.427***	0.486***
Financial support from children			0.136***
Emotional support from children			0.173***
Gender (reference group: female)			
Male		0.166	0.197*
Age		−0.057***	−0.058***
Education (reference group: no formal education)			
Elementary school graduate or below		0.757***	0.735***
Junior high school graduate		0.755***	0.715***
High school graduate or above		1.265***	1.206***
Marital status (reference group: not in a marital relationship)			
In a marital relationship		0.645***	0.629***
Hukou (reference group: non-agricultural hukou)			
Agricultural hukou		−0.332*	−0.370**
Personal income (log-transformed)		0.307**	0.295**
Self-assessment of health (reference group: very healthy)			
Very unhealthy		−6.282***	−6.170***
Somewhat unhealthy		−5.258***	−5.236***
Average		−3.521***	−3.516***
Somewhat healthy		−1.835***	−1.847***
Basic activity assessment		−0.211***	−0.216***
Daily activity assessment		−0.354***	−0.374***
Total number of living children		−0.119**	−0.060
Pension security		−0.283***	−0.263**
Social support		0.059***	0.049***
Internet usage (reference group: no use)			
Use		0.706***	0.570***
Community environment		0.014	−0.006
Social engagement		−0.156***	−0.156***
Work environment (reference group: very comfortable)			
Very poor		−0.628**	−0.653**
Fairly poor		−1.526***	−1.505***
Average		−0.943***	−0.916***
Fairly comfortable		−0.450*	−0.474*
Current work frequency (reference group: almost every day)			
Not participating		−1.707***	−1.744***
Several times a year		−1.663***	−1.744***
At least once a month		−1.513***	−1.555***
At least once a week		−1.642***	−1.634***
Retirement status (reference group: no)			
Yes		1.082***	1.070***
Constant	37.371**	47.647**	45.990**
Sample size	8,754	8,754	8,754
*R*^2^	0.025	0.300	0.308

* indicates *p* < 0.05, ** indicates *p* < 0.01, *** indicates *p* < 0.001.

Regarding core independent variables: Model 1 results indicate that, without controlling for variables, older adults individuals residing within the same city but at a distance from their children or living in their village/neighborhood committee demonstrated significantly higher mental health levels compared to those cohabiting with their children or residing locally, with a coefficient increase of 1.519 units (*p* < 0.001). This model preliminarily reveals a trend where older adults individuals with intergenerational residential distance within the same city exhibit superior mental health compared to those cohabiting or living in close proximity. After incorporating relevant control variables in Model 2, intergenerational residential distance continues to exert a stable positive influence on older population’s mental health (*p* < 0.001). For the older population, moderate intergenerational residential distance implies reduced generational friction and increased freedom in daily life (36), which contributes to improved mental health. Notably, increased geographical distance does not necessarily equate to psychological estrangement; children can effectively promote older population’s mental health through diversified forms of intergenerational support (18). Accordingly, Hypothesis H1 is validated. Regarding control variables: Based on Model 2’s statistical results, age, agricultural household registration (dummy variable), self-rated health (dummy variable), basic activity capacity, daily activity capacity, the total number of surviving children, pension security, social participation, work environment (dummy variable), and current work frequency (dummy variable) all exerted significant negative effects on older adults’ mental health. While educational attainment (dummy variable), marital status (dummy variable), personal income, social support status, internet usage (dummy variable), and retirement status (dummy variable) exerted significant positive effects. Due to space constraints, further elaboration is omitted here. Regarding mediating variables: Model 3 results indicate that, after controlling for intergenerational residential distance and other variables, both financial support and contact-based emotional support from children significantly positively influence older adults’ mental health (*p* < 0.001). Specifically, each additional unit of financial support from children increased older adults’ mental health by 0.136 units, while each additional unit of contact-based emotional support increased mental health by 0.173 units. This indicates that intergenerational support from children significantly influences the mental health of older parents, with contact-based emotional support having a stronger effect than financial support (0.173 > 0.136).

### 4.2 Mediation effect test

**Figure 1:**
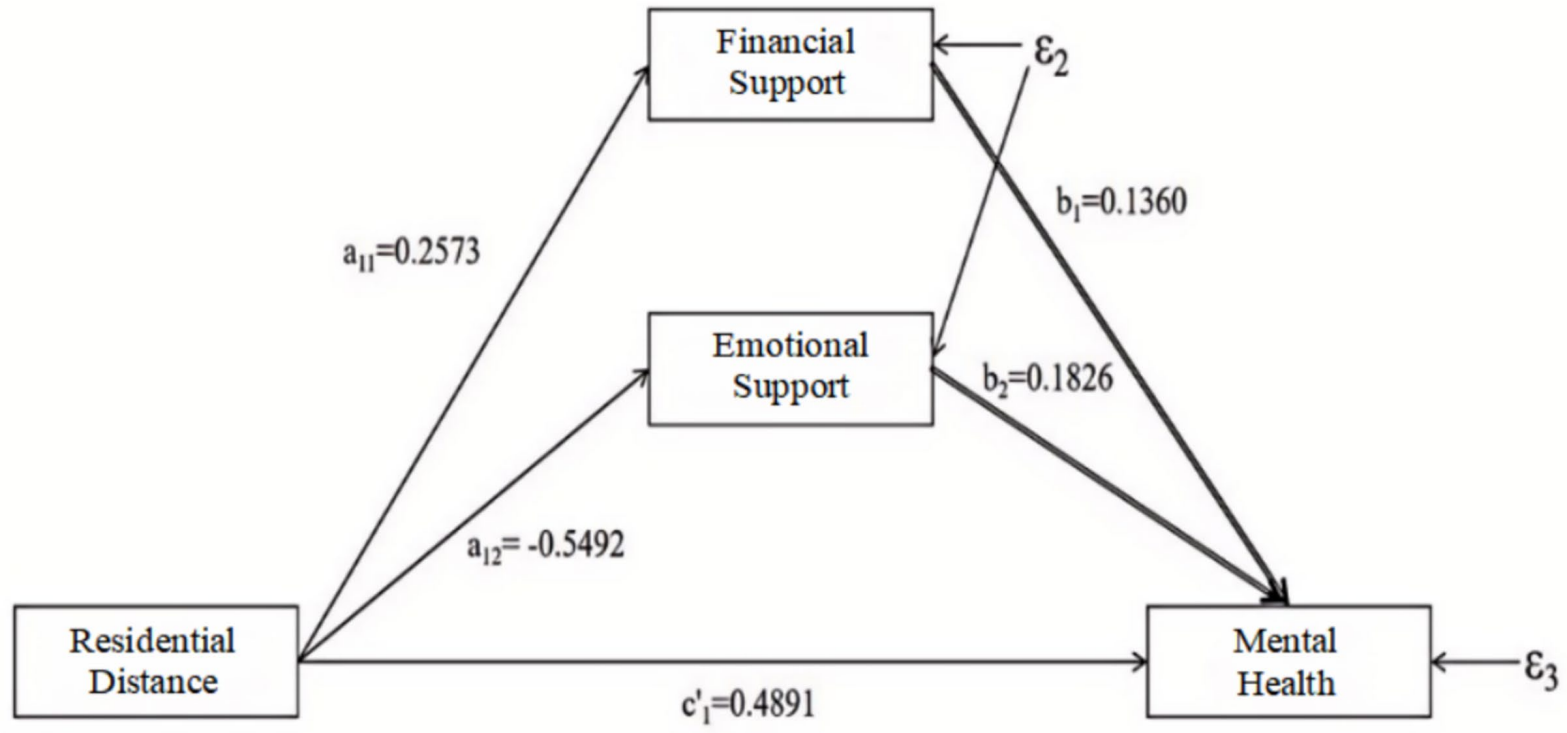
Mediated effect model diagram.

Among the older population, family support stands as one of the most crucial sources of social support, exerting a unique and profound influence on their mental health. This support encompasses not only material assistance but also emotional and spiritual sustenance. Based on this, this paper will further explore the mediating role of children’s support in the impact of intergenerational residential distance on the mental health of older adults. This analysis will be conducted using the OLS regression model and the SPSS Process plugin. Table 3 presents the results of 5,000 simulations conducted within a 95% confidence interval. Figure 1 illustrates the specific pathway of the mediating effect of upward intergenerational support between intergenerational residential distance and the mental health of older adults.

**Table 3:** Mediating effect test.

Effect type	Effect path	Effect value	SE	*P* value	Boot CI	
					LLCI	ULCI
Total effect	Intergenerational residential distance → mental health	0.4238	0.0933	0.0000	0.2410	0.6067
Direct effect	Intergenerational residential distance → mental Health	0.4891	0.0945	0.0000	0.3039	0.6743
Indirect effect	Intergenerational residential distance → Children’s financial/emotional support → mental health	−0.0653	0.0201	——	−0.1057	−0.0268
	Intergenerational residential distance → Children’s financial support → Mental health (IE_1_)	0.0350	0.0087	——	0.0192	0.0529
	Intergenerational residential distance → Children’s emotional support → Mental health (IE_2_)	−0.1003	0.0175	——	−0.1356	−0.0670
Mediated effect difference	IE_1_-IE_2_	0.1352	0.0191	——	0.0985	0.1738

1. The reference group for the independent variable “intergenerational residential distance” is “the household and the village/neighborhood committee.” 2. Results not output by the model are indicated by “——”; when no p-value is available, conclusions are primarily drawn based on confidence intervals.

Combining Figure 1 and Table 3 reveals that the path “intergenerational residential distance → children’s financial support & contact-based emotional support → mental health” constitutes the overall mediating effect pathway. The 95% Bootstrap confidence interval for this path is [−0.1057, −0.0268], which does not include zero. This indicates that the total mediating effect is significant, meaning that the overall indirect influence of generational residential distance on older adults’ mental health, mediated through children’s financial support and contact-based emotional support, is statistically significant.

Analysis of specific indirect effects for each mediating variable revealed: On one hand, in the pathway “intergenerational residential distance → children’s financial support → mental health,” the 95% Bootstrap confidence interval for the relative mediating effect was [0.0192, 0.0529], which did not include zero, indicating a significant effect. Specifically, compared to the reference group living with their children or within the same village/neighborhood committee, older adults residing at a distance of “within the same city” received an average of 0.2573 additional units of financial support from their children, which correspondingly increased their mental health levels by 0.0350 units. On the other hand, in the pathway “intergenerational residential distance → children’s contact-based emotional support → mental health,” the 95% Bootstrap confidence interval for the relative mediating effect is [−0.1356, −0.0670], which does not include zero, indicating a similarly significant effect. Data indicates that, compared to the reference group residing in their village/neighborhood committee, older adult individuals living with their children (in the same household) receive an average of 0.5492 fewer units of contact-based emotional support, resulting in a corresponding decrease of 0.1003 units in their mental health levels (Note: This logic implicitly involves comparative differences relative to “within the city” or other reference groups; it should be understood in conjunction with the original model settings). The original intent is preserved in the revised wording. Furthermore, the relatively direct effect of “intergenerational residential distance → mental health” is significant (c’1 = 0.4891, *p* < 0.001). This indicates that, after controlling for mediating variables, older adult individuals whose children reside within the same city still exhibit mental health levels 0.4891 units higher than those whose children live in the same household or village/neighborhood committee.

Regarding the direction of mediation, in the path of children’s financial support, the path coefficients are a11 = 0.2573, b1 = 0.1360, with an indirect effect value of a11*b1 = 0.0350. Since all coefficients and effect values are greater than zero, this indicates that children’s financial support exerts a positive mediating effect on the influence of intergenerational residential distance on older adults’ mental health, supporting hypothesis H₂a. In the path of children’s contact-based emotional support, the path coefficient a12 = −0.5492, b2 = 0.1826, and the indirect effect value a12*b2 = −0.1003. Since the indirect effect value is negative, it indicates that children’s contact-based emotional support mediates negatively in the relationship between intergenerational residential distance and older adults’ mental health. Thus, hypothesis H2b is supported.

Comparing the effect sizes of the two mediating pathways reveals that: “Intergenerational residential distance → Children’s financial support → Mental health” yielded an effect size of 0.0350 (95% CI [0.0192, 0.0529]), while “Intergenerational residential distance → Children’s contact-based emotional support → Mental health” produced an effect size of −0.1003 (95% CI [−0.1356, −0.0670]). Although opposite in direction, the absolute value of the effect transmitted through contact-based emotional support was significantly greater than that of financial support (|-0.1003| > 0.0350). The difference test between the two effects showed a significant difference in effect size (*Δ* = 0.1352, 95% CI [0.0985, 0.1738]). This indicates that the shift in mental health levels resulting from children moving from residing within the same household or village/neighborhood committee to residing within the same city is primarily mediated by a reduction in contact-based emotional support from children (release of negative effects) rather than an increase in financial support. In other words, compared to financial support, contact-based emotional support from children exerts a stronger mediating effect on the impact of intergenerational residential distance on older adults’ mental health.

### 4.3 Robustness test

To ensure the reliability of regression results, this study conducted the following robustness tests:

(1) Handling extreme values. OLS regression is relatively sensitive to extreme/outlier values, and results may be influenced by one or two outliers. This study opted for right-truncation at the 1% level for the continuous dependent variable “mental health level,” replacing the extreme values at the 1st and 99th percentiles with the 1st and 99th percentiles, respectively. Regression analysis was re-conducted using the trimmed data, with results presented in Table 4, Model 4. The results indicate that the coefficients and significance levels of key variables remained essentially unchanged compared to the baseline regression, confirming the robustness of the findings.
(2) Adjusting the set of control variables. This study examined whether the findings were sensitive to the selection of control variables by adjusting their set. The regression results are presented in Table 4, Model 5. First, Model 5 removed the community environment variable from the baseline model, and the core conclusions remained unchanged. Second, Model 6 further added the control variable “willingness to receive care” to Model 5, and the effect of the independent variable remained significant. This indicates that the study’s findings are not sensitive to the selection of control variables.
(3) Subsample analysis. We selected subsamples based on the key variables of age and urban/rural status, performed full-model regression analyses on each of the four subsamples, and compared the direction and significance of the independent variables across the subsamples. First, the older population was divided into two groups: younger older population (ages 60–69) and middle-to-older older population (ages 70 and above). As shown in Models 8 and 9 in Table 4, the direction of the independent variables’ effects was consistent and significant across all subsamples, indicating robust conclusions. Second, the urban and rural samples were separated. As shown in Models 10 and 11 in Table 4, certain differences existed between the two groups. The main findings are summarized as follows.

**Table 4:** Robustness test.

Variables	Mental Health			
	Model 4	Model 5	Model 6	Model 7
Intergenerational residential distance (reference group: this household and this village/neighborhood committee)				
This prefecture-level city	0.479***	0.486***	0.443***	0.202***
Financial support from children	0.136***	0.135***	0.145***	0.050***
Emotional support from children	0.170***	0.173***	0.204***	0.075***
Control variable	Control	Control	Control	Control
Constant	45.933**	46.003**	45.309**	——
Sample size	8,754	8,754	7,933	8,754
*R*^2^ or pseudo *R*^2^	0.306	0.308	0.316	0.272
Intergenerational residential distance (reference group: this household and this village/neighborhood committee)				
This prefecture-level city	0.363*	0.530***	0.160***	0.062
Financial support from children	0.169***	0.109***	0.005	0.042***
Emotional support from children	0.042	0.257***	0.036***	0.045***
Control variable	Control	Control	Control	Control
Constant	46.421***	46.419***	1.411***	3.500***
Sample size	3,390	5,364	5,132	3,622
*R*^2^ or pseudo *R*^2^	0.291	0.287	0.246	0.290

* indicates *p* < 0.05, ** indicates *p* < 0.01, *** indicates *p* < 0.001.

Regarding the independent variables, having children residing in the same city (compared to living in the same village or under the same roof) significantly improves the mental health of rural older adults, but this effect is not significant for urban older adults. There are two main reasons for this urban–rural disparity: first, public resources are relatively scarce in rural areas, leading older adults to rely more heavily on family support. Living at a “moderate distance” from children facilitates daily visits and emotional interaction, alleviating depressive symptoms; it also allows the older population to avoid the friction associated with intergenerational cohabitation, maintaining their autonomy and cognitive engagement, thereby providing a significant protective effect on mental health. Second, urban older population has access to more extensive community services and social networks, resulting in lower dependence on their children; consequently, the additional benefits of intergenerational living distance on their mental health are relatively limited.

Regarding mediating variables, financial support from children significantly improved the mental health of urban older adults but had no significant effect on rural older adults; in contrast, contact-based emotional support had a significant positive impact on the mental health of older adults in both urban and rural areas, with a stronger effect observed among urban older adults. Overall, whether in urban or rural areas, the psychological improvement effect of contact-based emotional support is significantly greater than that of financial support. The main reasons for this phenomenon are as follows: On the one hand, due to factors such as children being away from home for extended periods, the older population generally face issues of “emotional deprivation” and accumulated loneliness. Compared to financial support, which is often viewed as a daily obligation or a form of filial piety, children’s emotional responses, expressions of care, and regular communication make the older population feel more valued, thereby effectively alleviating depressive symptoms and enhancing life satisfaction. On the other hand, as society evolves, the older population’s need for contact-based emotional support has been on the rise, even surpassing their reliance on purely financial assistance. Consequently, statistical findings consistently show that contact-based emotional support exerts a more significant psychological protective effect than financial support.

(1) Subsample analysis. Subsamples were selected based on the key variable of age, dividing older adults into younger seniors (60–69 years) and older seniors (70 years and above). Full model regressions were conducted for each subsample to compare the direction and significance of the independent variable’s effect across subsamples. Results are shown in Models 9 and 10 of Table 4. The effect directions of the independent variables were consistent and significant across all subsamples, confirming the robustness of the conclusions.
(2) Propensity score matching was employed to further estimate the impact of intergenerational residential distance on older adults’ mental health.

On the one hand, intergenerational residential distance may be determined by initial conditions such as other demographic or socioeconomic characteristics. In other words, the “intergenerational residential distance” for each sample is not a random variable but rather self-selected. On the other hand, a bidirectional causal relationship may exist between generational living distance and older adults’ mental health. Therefore, examining this relationship through an OLS benchmark regression may introduce endogeneity bias. To address this issue, this study employs Propensity Score Matching (PSM) to further estimate the relationship between generational living distance and older adults’ mental health. Table 5 presents the ATT estimates of generational living distance’s impact on older adults’ mental health. The average treatment effects from the k-nearest neighbors matching (*k* = 4) and radius matching (*r* = 0.01) methods are comparable to the OLS regression results. Moreover, the ATT values from both methods are statistically significant at the 1% level. This finding indicates that, when accounting for sample selection bias, older adults whose children reside within the same household or village/neighborhood neighborhood exhibit higher mental health levels. Thus, the OLS regression model results are relatively reliable, supporting H1.

**Table 5:** ATT estimates.

Variable	Matching method	ATT	SE	*T*-value
This household and this village/neighborhood committee	*k*-nearest neighbors matching (*k* = 4)	0.54	0.125	4.33***
	Radius matching (*r* = 0.01)	0.464	0.115	4.03***

* indicates *p* < 0.05, ** indicates *p* < 0.01, *** indicates *p* < 0.001.

**Figure 2:**
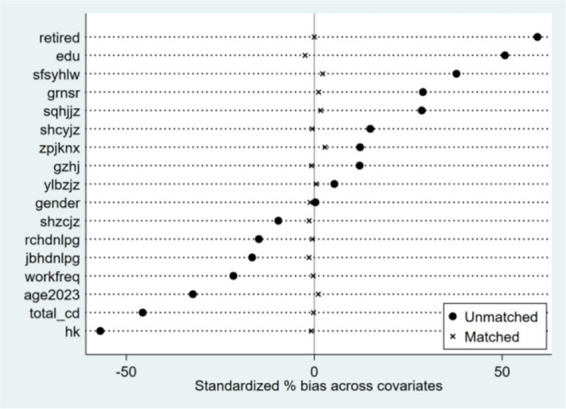
Standardized biases.

**Figure 3:**
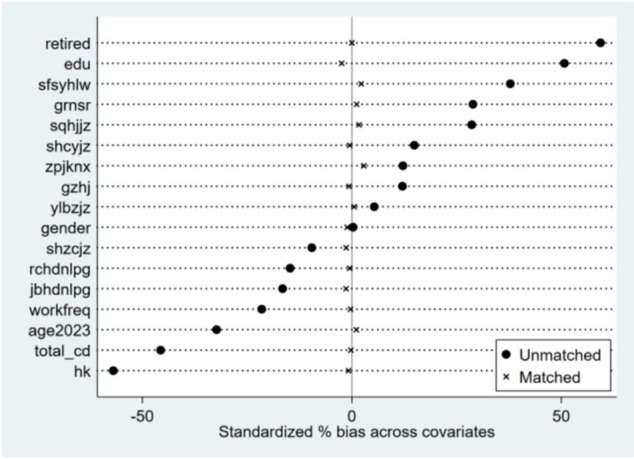
Standardized biases.

**Figure 4:**
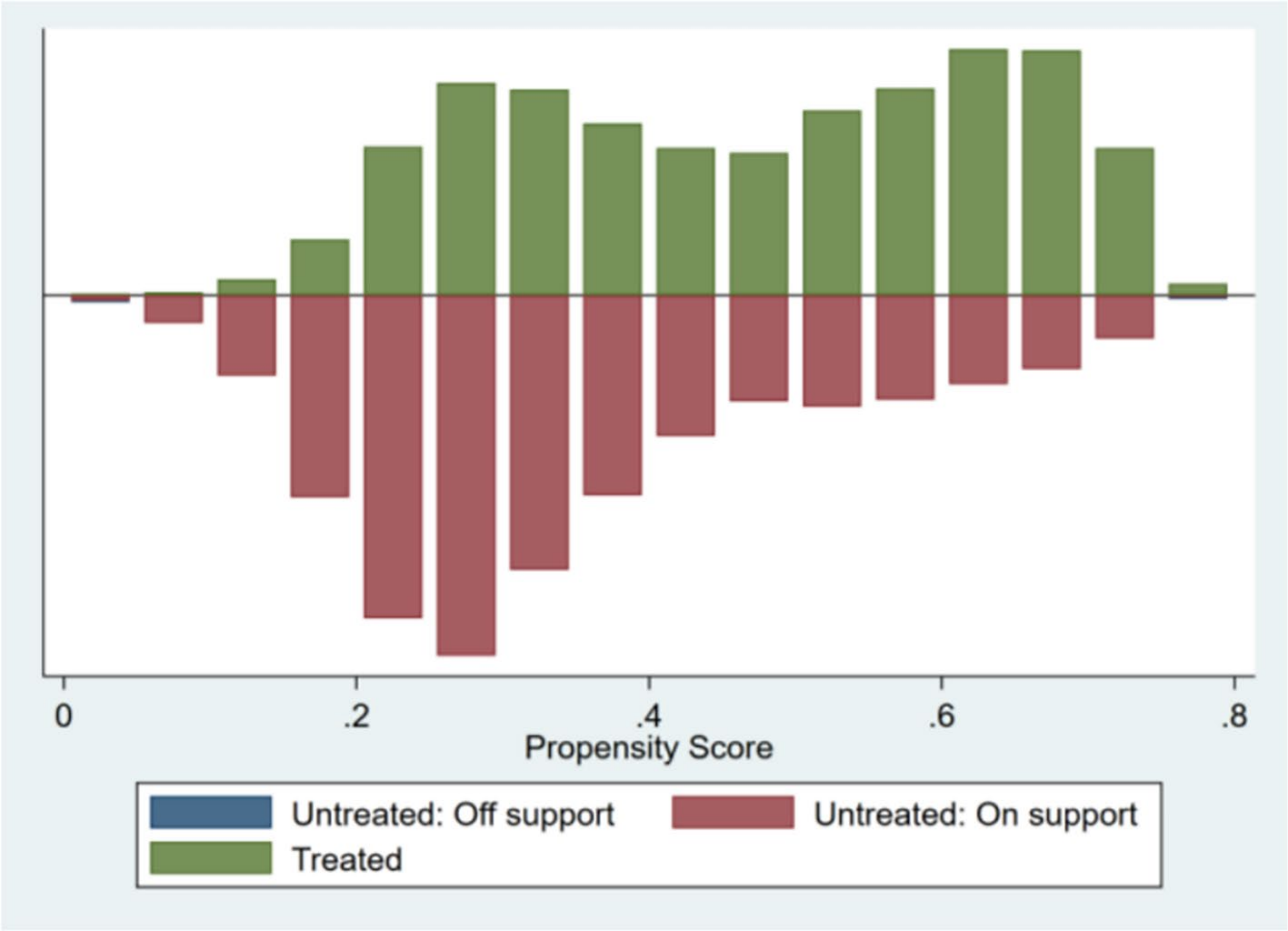
Common support of propensity scores.

**Figure 5:**
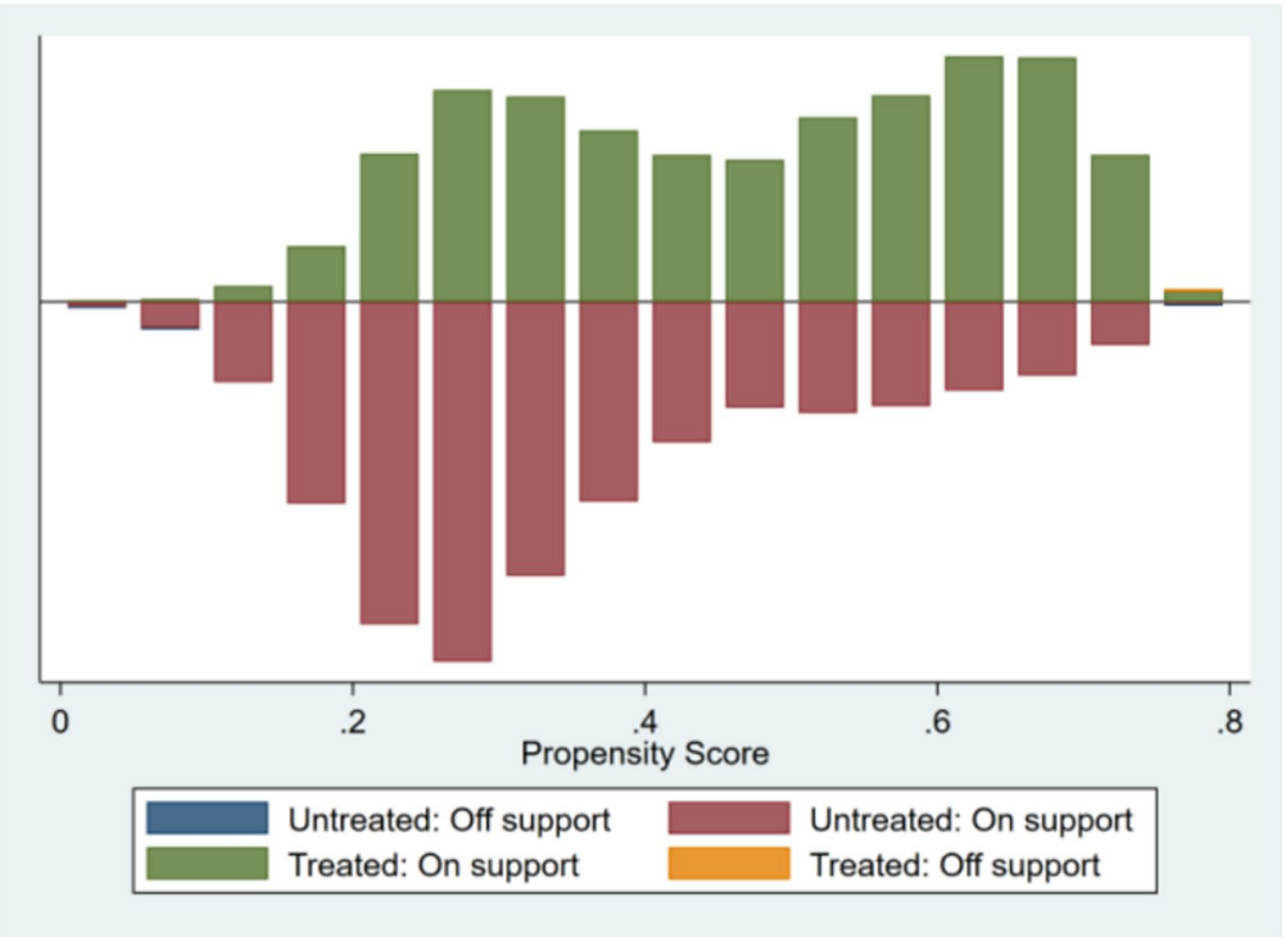
Common support of propensity scores.

Figures 2, 3 present the changes in standardized deviations for each variable before and after “neighborhood matching” and “radius matching,” respectively, in the average treatment effect estimates where the experimental group is “this prefecture-level city” and the control group is “this household and this village/neighborhood committee.” This provides a more intuitive illustration of the balance achieved through matching. Compared to the unmatched state, the standardized errors for the vast majority of variables are closer to zero after matching. This indicates that, overall, the matching effect is relatively good. Figures 4, 5 reveal that in the average treatment effect estimates where the experimental group is “this prefecture-level city” and the control group is “this household and this village/neighborhood committee,” most observations fall within a common range of values. Consequently, only a small number of cases are lost during propensity score matching, indicating the effectiveness of both “nearest neighbor matching” and “radius matching.”

## 5 Conclusions and discussion

### 5.1 Main conclusions

Against the backdrop of accelerated modernization and increasing social mobility, profound transformations have occurred in family residential patterns and intergenerational attitudes, with growing disparities emerging between parents and their children in lifestyle choices and value orientations. In this context, the distance of intergenerational cohabitation has become a key factor influencing the mental health of older adults. Guided by the “Intergenerational Solidarity-Conflict Theory” and the “Social Support Theory,” this study empirically examines the impact of intergenerational residential distance on older adults’ mental health using the 2023 CLASS data. Key findings are as follows:

First, compared to living with children or within the same village/neighborhood committee, older adults whose children reside within the same city exhibit significantly higher mental health levels, validating Hypothesis H1. This finding corroborates previous research on intergenerational conflict: the negative effects of cohabitation or proximity may offset or even outweigh positive benefits, ultimately harming older adults’ mental health (37). Therefore, intergenerational cohabitation or proximity may not represent the optimal residential arrangement for enhancing older adults’ mental health. Maintaining a certain distance from children allows older adults to preserve relative independence—fulfilling their value pursuit of autonomous living (26)—while effectively reducing intergenerational friction and downward pressure on children (38). simultaneously ensuring flexibility and convenience for children to provide upward support, thereby promoting better mental health outcomes among the older population (39). It is important to note that our binary measure (same household/village vs. same city) does not capture fine-grained gradations of distance; therefore, while our findings are consistent with the idea of a “moderate distance” advantage, they do not directly test it. Future research with more refined distance measures is needed to confirm this interpretation.

Second, children’s financial support plays a positive mediating role in the process where intergenerational residential distance affects older adults’ mental health, supporting hypothesis H2a. Existing research indicates that children who do not live with their parents often provide higher levels of financial support (40). Further research indicates that increased intergenerational residential distance may stimulate compensatory motivation among children: to make up for the lack of daily care and emotional communication caused by spatial living apart, children tend to increase material support as compensation (25). In other words, as residential distance increases, children strengthen intergenerational bonds through enhanced financial transfers to mitigate the absence of companionship and care, thereby improving the mental health of the older population. This mechanism aligns strongly with the empirical findings of this study.

Third, children’s contact-based emotional support played a negative mediating role in the process where intergenerational residential distance affects older adults’ mental health, validating Hypothesis H2b. Research indicates that closer residential proximity correlates with a higher frequency of emotional exchange and interaction between parents and children, enabling older adults to more readily obtain emotional comfort and psychological peace (41). However, increased mobility among children reduces face-to-face communication frequency. Expanding physical distance often accompanies emotional estrangement, diminishing the efficiency of emotional comfort transmission and adversely affecting the physical and mental health of the older population (42).

Fourth, compared to children’s financial support, their contact-based emotional support exhibits a stronger mediating effect in the relationship between intergenerational residential distance and older adults’ mental health. Numerous studies indicate that among various forms of intergenerational support, contact-based emotional support has the most significant positive impact on older adults’ mental health (27). The older population stage is often accompanied by social marginalization and a diminished sense of existence, making mental health issues particularly prominent. At this stage, contact-based emotional support from children can reinforce older adults’ sense of self-worth through feeling needed, reshape their sense of family belonging and life meaning, thereby enhancing life satisfaction and happiness, and effectively alleviating depression and anxiety. Furthermore, positive emotional interaction not only helps delay cognitive decline, but moderate contact-based emotional support can also partially compensate for insufficient financial support, playing a more crucial role in psychological adaptation.

Fifth, while our mediation analysis empirically confirms that financial support increases and contact-based emotional support decreases with greater residential distance, the underlying social processes warrant deeper theoretical elaboration. Cultural norms of filial piety in China add nuance. For many Chinese families, living apart is sometimes framed as a necessary sacrifice for children’s career advancement, and parents may take pride in their children’s success—a feeling that can partially offset the loss of daily companionship. However, this pride-based compensation is not captured by our financial or contact-frequency measures. Future research using mixed methods (e.g., in-depth interviews or communication diaries) is needed to unpack these qualitatively distinct mechanisms.

### 5.2 Policy implications

Our findings carry several concrete implications for policy and practice:

First, support “separate but close” living arrangements. For older adults with adequate self-care ability, policies should facilitate residential patterns where children live within the same city but not in the same household. This can be encouraged through housing subsidies for multi-generational proximity (e.g., preferential mortgage terms for parents and children buying flats in the same district), improved public transportation connecting different districts, and portable healthcare coverage that allows older adults to access medical services near their children’s homes without administrative hassle.

Second, prioritize emotional connection over financial transfers. Our mediation analysis shows that contact-based emotional support has a stronger impact on mental health than financial support, and that distance reduces this support. Therefore, policy interventions should go beyond cash transfers (e.g., pensions, child-to-parent remittance incentives) and actively promote emotional bonding. Specific measures include:

- Launching a national “Digital Companionship” program that provides free or subsidized tablets/phones and digital literacy training for older adults, enabling regular video calls with distant children.
- Encouraging employers to offer “filial leave” (paid days off) for employees to visit parents living in other cities, modeled after policies in some Chinese provinces (e.g., Fujian’s “care leave for older adult parents”).
- Supporting community-based “intergenerational communication workshops” that teach families how to maintain meaningful conversations across distance, focusing on quality rather than frequency.

Third, tailor policies to urban–rural differences. For rural older adults (who show stronger benefits from same-city residence), governments should invest in village-level mutual aid networks and day-care centers to compensate for the absence of daily child contact. For urban older adults, where the distance effect is weaker, policies should focus on expanding community social activities, senior clubs, and volunteer visitation programs that offer alternative sources of emotional support independent of children.

Finally, address the limitations of our findings in policy design. Because our study cannot fully rule out reverse causality, policies encouraging “separate living” should be piloted with careful evaluation rather than universally recommended. Decision-makers should also recognize that our measure of emotional support is based on contact frequency; policies aiming to improve emotional support should emphasize communication quality, not just frequency.

### 5.3 Limitations and future directions

This study has the following limitations, which should be addressed in future research:

First, a central limitation concerns the measurement of intergenerational residential distance. Our primary theoretical claim pertains to the benefits of a “moderate distance.” However, due to sample size constraints in the original survey, we collapsed eight original categories into a binary variable: (0) same household or village/neighborhood committee, and (1) within the same city. This dichotomization conflates substantively different distances (e.g., same neighborhood vs. same district) and cannot capture the non-linear or threshold effects implied by the concept of moderate distance. Consequently, our empirical findings should be interpreted as a comparison between “very close” and “same-city” arrangements, rather than as a direct test of a graded “moderate distance” effect. We have tempered our language throughout the manuscript accordingly and urge future research to employ continuous or multi-category distance measures to more directly test the moderate-distance hypothesis. Furthermore, our measure of emotional support relies on the frequency of contact and visits, which does not capture the quality, warmth, or perceived adequacy of emotional exchanges. Future studies should incorporate validated scales of emotional support quality (e.g., the Emotional Support subscale of the MOS Social Support Survey) to disentangle the distinct effects of contact frequency versus supportive quality.

Second, regarding data sources and structure. The data in this study are based on self-reports from older adults, which may introduce subjective biases, such as underestimating the level of support from children or overestimating one’s own mental health status. If data from the children’s perspective can be incorporated for cross-validation in future studies, the reliability of the data will be significantly enhanced. Furthermore, this study relies solely on cross-sectional data for analysis, making it difficult to capture the dynamic trends in the impact of intergenerational living distance on mental health. Future research could utilize multi-wave longitudinal data, such as the CLASS study, to construct panel or fixed-effects models. This would allow for the control of individual heterogeneity and time trends, thereby enhancing the robustness of causal inferences.

Third, this study did not include variables reflecting family structural heterogeneity, such as the gender composition of children, marital status, or residence abroad, due to limitations in the CLASS dataset’s recording of individual characteristics of children. This omission may overlook the differentiated mechanisms of intergenerational support across different family structures, such as the burden-sharing effect in families with multiple children or the pressure of being the sole dependent in families with an only child. Future research that integrates more detailed child-level data will help deepen our understanding of the heterogeneous mechanisms of intergenerational support.

Fourth, despite our use of Propensity Score Matching to balance observable covariates, the possibility of unobserved selection bias remains. For instance, older adults with more resilient personalities or higher prior life satisfaction may be more inclined to choose independent living, and such traits are often unmeasured in survey data. Conversely, children who have experienced high-quality early relationships with their parents might be more likely to maintain close residential proximity while also promoting parental mental health through other channels. Our PSM approach cannot rule out these alternative explanations because it only conditions on observed variables. Therefore, while our findings are robust to observable confounders, they should be interpreted as associations rather than pure causal effects. Future research could employ instrumental variable strategies (e.g., using children’s job location or housing prices as instruments) or longitudinal designs with individual fixed effects to better disentangle causality from selection.

## Data Availability

The data used in this study are from the China Longitudinal Aging Social Survey (CLASS), designed and implemented by the China Institute of Health, Renmin University of China. The data are available upon reasonable request from the survey team via the official application portal: http://jkzgyjy.ruc.edu.cn/sjzy/CLASSsjsq/ade1ba3977354553a798c8f10221c2ad.htm.
